# HMGB1: A New Target for Ischemic Stroke and Hemorrhagic Transformation

**DOI:** 10.1007/s12975-024-01258-5

**Published:** 2024-05-14

**Authors:** Jiamin Li, Zixin Wang, Jiameng Li, Haiping Zhao, Qingfeng Ma

**Affiliations:** Department of Neurology and Cerebrovascular Diseases Research Institute, Xuanwu Hospital, Capital Medical University, 45 Changchun Street, Beijing, China

**Keywords:** HMGB1, Stroke, Hemorrhagic transformation, tPA, Neuroinflammation, Microglia, Neutrophil, Macrophage, T cell

## Abstract

Stroke in China is distinguished by its high rates of morbidity, recurrence, disability, and mortality. The ultra-early administration of rtPA is essential for restoring perfusion in acute ischemic stroke, though it concurrently elevates the risk of hemorrhagic transformation. High-mobility group box 1 (HMGB1) emerges as a pivotal player in neuroinflammation after brain ischemia and ischemia–reperfusion. Released passively by necrotic cells and actively secreted, including direct secretion of HMGB1 into the extracellular space and packaging of HMGB1 into intracellular vesicles by immune cells, glial cells, platelets, and endothelial cells, HMGB1 represents a prototypical damage-associated molecular pattern (DAMP). It is intricately involved in the pathogenesis of atherosclerosis, thromboembolism, and detrimental inflammation during the early phases of ischemic stroke. Moreover, HMGB1 significantly contributes to neurovascular remodeling and functional recovery in later stages. Significantly, HMGB1 mediates hemorrhagic transformation by facilitating neuroinflammation, directly compromising the integrity of the blood–brain barrier, and enhancing MMP9 secretion through its interaction with rtPA. As a systemic inflammatory factor, HMGB1 is also implicated in post-stroke depression and an elevated risk of stroke-associated pneumonia. The role of HMGB1 extends to influencing the pathogenesis of ischemia by polarizing various subtypes of immune and glial cells. This includes mediating excitotoxicity due to excitatory amino acids, autophagy, MMP9 release, NET formation, and autocrine trophic pathways. Given its multifaceted role, HMGB1 is recognized as a crucial therapeutic target and prognostic marker for ischemic stroke and hemorrhagic transformation. In this review, we summarize the structure and redox properties, secretion and pathways, regulation of immune cell activity, the role of pathophysiological mechanisms in stroke, and hemorrhage transformation for HMGB1, which will pave the way for developing new neuroprotective drugs, reduction of post-stroke neuroinflammation, and expansion of thrombolysis time window.

## Introduction

In China, stroke exhibits high incidence, recurrence, disability, and mortality rates, placing the country at the forefront globally in terms of stroke-related deaths and disability-adjusted life years [1]. Analyses for the Global Burden of Disease Study 2019 found that there were 12·2 million (95% UI 11·0–13·6) incident strokes globally and 3·94 million (95% UI 3·43–4·58) incident strokes in China in 2019, with the largest increase in ischemic stroke incidence in China from 1990 to 2019 at 22.5% (21.3 ~ 24.5) [1, 2]. Ultra-early application of rtPA (recombinant tissue plasminogen activator) is essential for the recovery of perfusion to ischemic brain tissue in acute cerebral infarction. Still, it also increases the risk of hemorrhagic transformation, which may result in deterioration of neurological function. Identifying early predictors of hemorrhagic transformation can help reduce its incidence and severity. Stroke-induced inflammation and activation of pro-inflammatory mediators have been the focus of studies on the mechanisms of ischemic stroke-induced brain injury, with neuroinflammation leading to apoptosis, blood–brain barrier disruption, cerebral edema, and hemorrhagic transformation in the acute phase while supporting tissue repair and functional recovery in the late stage [3].

High-mobility group box 1 (HMGB1) is a highly conserved non-histone DNA-binding protein in the nucleus that can be released into the extracellular environment to mediate inflammatory responses [4]. Damage-associated molecular pattern (DAMP) is passively released or actively secreted from cells, activates the innate immune system, and is involved in the direct and rapid activation of the inflammatory response after stroke onset. HMGB1 is a typical DAMP molecule that, in addition to being involved in neuroinflammation, has been found to mediate thrombosis, atherosclerosis, disruption of the blood–brain barrier, and, at a later stage, neurovascular remodeling and stroke recovery [5–7]. The complex role of HMGB1 in ischemic stroke and the mechanism of hemorrhagic transformation need to be further investigated and provide potential targets for neuroprotective strategies after stroke.

In this review, we elucidate the structural and redox properties, release mechanisms, receptors, and complex roles of HMGB1 in ischemic stroke, focusing on the part of HMGB1 with related neuroglia and immune cells and in hemorrhagic transformation. Furthermore, we have summarized therapeutic strategies targeting HMGB1, aiming to establish HMGB1 as a novel target for the treatment and prognostic prediction of ischemic stroke and hemorrhagic transformation. This approach could potentially serve as a basis for identifying individuals at risk of hemorrhagic conversion and for the adjunctive use of HMGB1 inhibitors in thrombolytic therapy to reduce the incidence of such transformations.

## Structure and Redox Properties of HMGB1

**Fig. 1:**
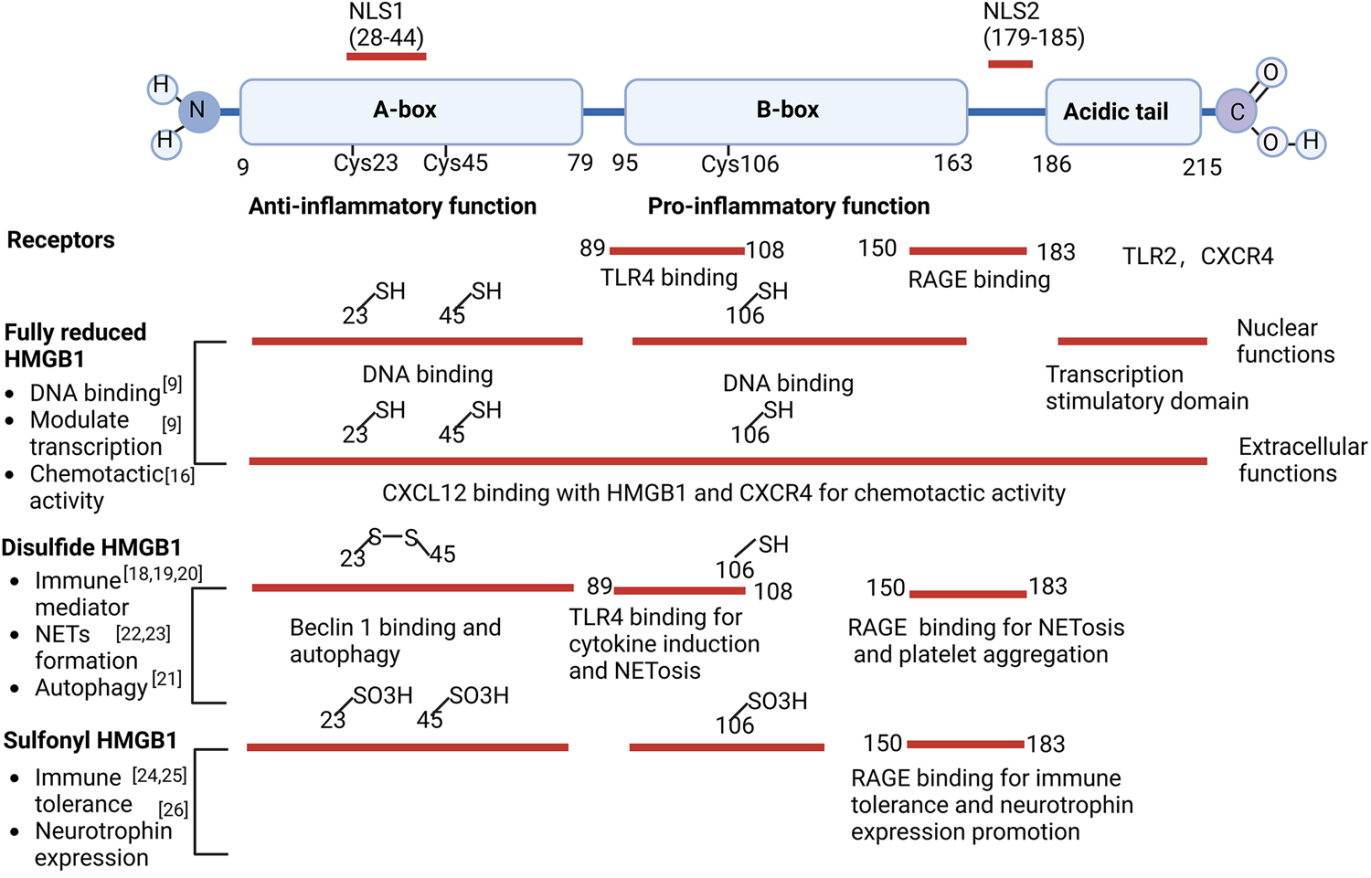
Structure, function, and redox properties of HMGB1. HMGB1 includes three structural domains in an anti-inflammatory A, a pro-inflammatory B boxes, and a 30 amino acid long c-segment tail. HMGB1 mediates the immune response by interacting with receptors such as toll-like receptor 4 (C89-C108) and late glycosylation end product-specific receptor (C150-C183). The redox state of HMGB1 is essential for the role of HMGB1 in regulating pathophysiological processes like inflammatory responses, autophagy, NET formation, and neurotrophin expression promotion

HMGB1 is a non-histone DNA-binding and amphipathic protein containing 215 amino acid residues, including three structural domains in a l-type arrangement of positively charged A and B boxes and a 30 amino acid long negatively charged c-segment tail [8, 9] (Fig. 1). The A box is associated with anti-inflammatory effects. In contrast, the B box is essential for cytokine induction and cell differentiation, and the c-terminal tail is related to regulating the DNA binding affinity of HMGB1 and enhancing the anti-inflammatory effects of the A box [10–13]. HMGB1 contains two nuclear localization signals, NLS1 (28–44 amino acids) and NLS2 (179–185 amino acids), as well as a nuclear export signal in the DNA-binding region (NES) [14], which contributes to the sublocalization of HMGB1 between the cytoplasm and nucleus.

The redox state of HMGB1 is essential for the role of HMGB1 in regulating inflammatory responses and autophagy. Redox modifications can occur at cysteine residues 23, 45, and 106, which regulate the functional properties of HMGB1 [15]. All three cysteines are present in the fully reduced state together with sulfhydryl residues in resting cells, and nuclear HMGB1 tends to be present in the fully reduced form [9]. Upon extracellular release, the all-sulfhydryl state of HMGB1 synergizes with the chemokine CXCL12 (C-X-C motif chemokine ligand 12) to form chemotactic heterocomplexes [16]. It binds synergistically to the CXCL12 chemokine receptor CXCR4(C-X-C chemokine receptor type 4), which contributes to the chemotactic activity of HMGB1 [16]. It was found that cysteine is not required for chemotactic activity, and serine replaces all of them to be more effective at recruiting leukocytes in vivo than wild-type HMGB1 [17]. An increase in oxygen free radicals leads to the formation of disulfide bonds on cysteine residues 23 and 45, and the cysteine residue 106 in the reduced state is required for HMGB1 to exert its pro-inflammatory action [18]. Disulfided HMGB1 can interact with the TLR4 (Toll-like receptor 4) receptor to activate NF-kB (nuclear factor kappa-B) signaling in microglia/macrophages to stimulate inflammatory responses and increase NMDA (N-methyl-D-aspartate)-induced neuronal cell death [19, 20]. However, it has been shown that the disulfide bond between the C23 and C45 of HMGB1 is required to bind to Beclin 1 to maintain the autophagic process, thereby enhancing cell survival in response to cellular stress [21]. The disulfide bond between C23 and C45 of HMGB1 disulfated HMGB1 also significantly increases platelet aggregation and promotes NETs (neutrophil extracellular traps) formation, which plays a vital role in thrombosis [22, 23]. All cysteine residues in HMGB1 can also be oxidized by sustained ROS (reactive oxygen species) and RNS (reactive nitrogen species) released from mitochondria of apoptotic cells and fully oxidized sulfonylated HMGB1 induces immune tolerance via the Rage pathway [24, 25]. In addition, oxidized HMGB1 was found to promote brain recovery by promoting the expression of neurotrophic factors [26]. The effects of different redox forms of HMGB1 on the complex roles of neuroimmunity and functional recovery in ischemic stroke at different times need further investigation.

## Release Mechanism of HMGB1

**Fig. 2:**
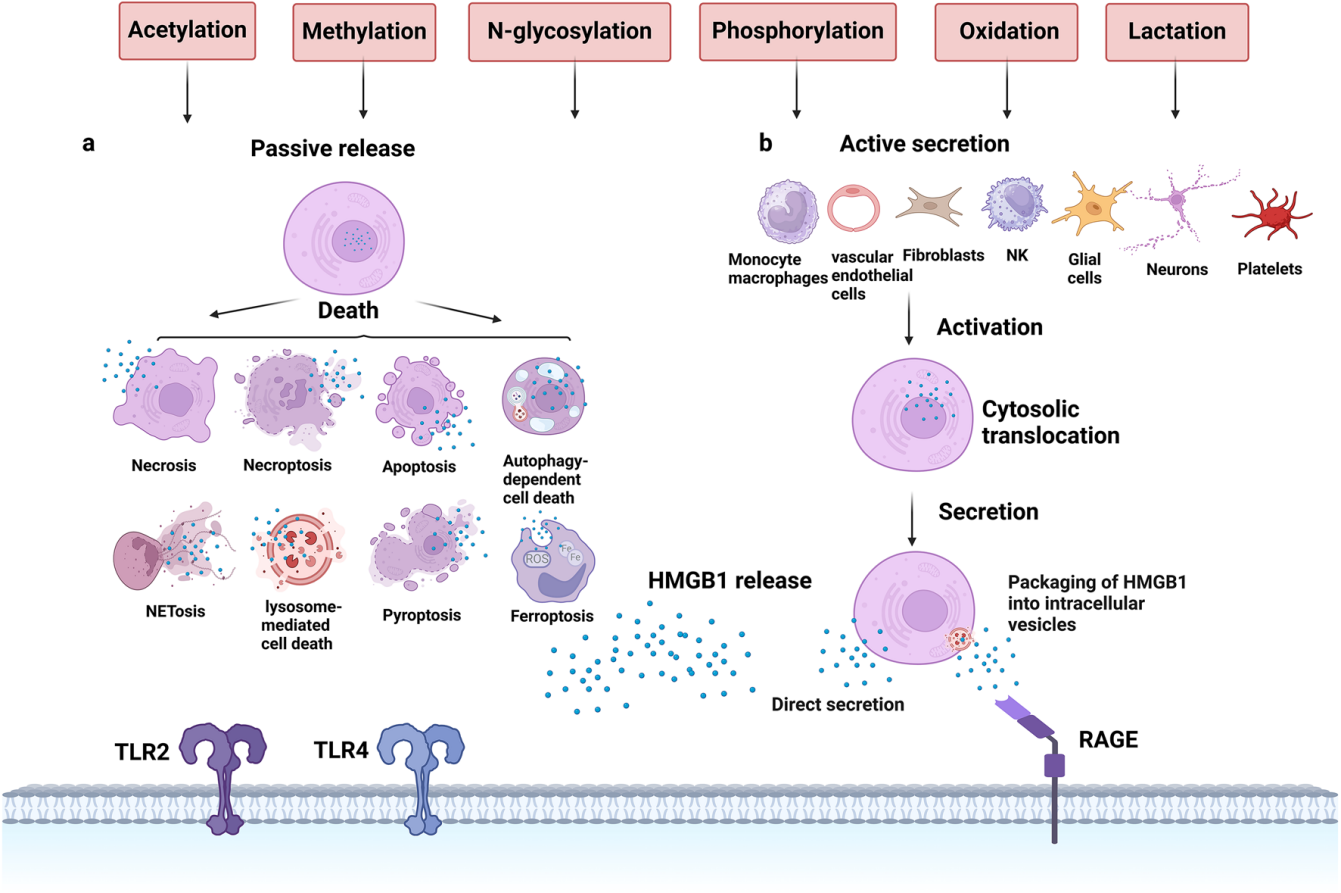
Release mechanism of HMGB1. HMGB1 release is regulated by various factors, including acetylation, methylation, N-glycosylation, phosphorylation, oxidative, and lactation. HMGB1 can be passively released after cell death and actively secreted, including direct secretion of HMGB1 into the extracellular space and packaging of HMGB1 into intracellular vesicles

HMGB1 release includes passive release in early post-stroke cell necrosis and active secretion in the late phase of stroke [27]. The secretion and release of HMGB1 are regulated by various factors, including acetylation, methylation, N-glycosylation, phosphorylation, and oxidation [14] (Fig. 2). Post-translational modifications of nuclear localization sequences (NLS) or nuclear export sequences (NES) affect the transfer of proteins from the nucleus to cytoplasmic accumulation and subsequent release, with the most studied mechanism in HMGB1 being lysine hyperacetylation within the NLS site [28, 29]. The acetylation of lysine regulates the cytoplasmic accumulation of HMGB1 through the co-determination of histone acetylase (HATS) and histone deacetylase (HDAC) [29]. Recent studies have explored the lactated modification of HMGB1, where lactated/acetylated HMGB1 is released from macrophages via exosomal secretion, thereby increasing endothelial permeability [30]. The effect of lactate-modified HMGB1 on the blood–brain barrier and hemorrhagic transformation needs further study.

Under these chemical modifications, the two specific forms of secretion of HMGB1 are as follows. HMGB1 can be passively secreted after cell death (such as necrosis, necroptosis, apoptosis, NETosis, lysosome-mediated cell death, pyroptosis, autophagy-dependent cell death, and ferroptosis) in response to various stimuli stimulation especially oxidative stress [14]. Active forms of secretion include direct secretion of HMGB1 into the extracellular space and packaging of HMGB1 into intracellular vesicles (such as lysosomes or autophagosomes), followed by release of HMGB1 into the extracellular after fusion of the vesicles with the cytoplasmic membrane [14].

After a stroke or other stressful injury, active secretion of HMGB1 was observed in activated monocyte macrophages, vascular endothelial cells, fibroblasts, NK cells, and glial cells [27]. When HMGB1 is secreted from necrotic cells or its secretion by activated macrophages, it induces the recruitment of inflammatory cells and mediates signaling between natural killer cells (NK cells), dendritic cells (DCs), T cells, and macrophages, thereby inducing positive feedback and exacerbating inflammatory injury [25]. In addition to the passive release of HMGB1 from neurons, recent studies have shown that stimulated peripheral sensory neurons actively transfer nuclear HMGB1 to the cytoplasm, where it is eventually released at nerve endings, stimulating the release of proinflammatory factors and exacerbating neuroinflammation in tissues. However, there are still fewer studies on the mechanism of the effect of active neuronal HMGB1 release on neuroinflammation in the central nervous system after stroke and the role of ablation of neuronal HMGB1 on ischemic brain tissue [31, 32]. Platelets are also an essential source of active HMGB1 secretion, and HMGB1 is critical in the acute phase of stroke to produce neutrophil extracellular traps(NETs) that exacerbate worsening post-stroke outcomes [33]. HMGB1 promotes monocyte accumulation and activation via the receptor for advanced glycosylation end products (RAGE) and Toll-like receptor 2 (TLR2). It facilitates RAGE-mediated formation of prethrombotic neutrophil extracellular traps (NETs), where the combined effects of coagulation and inflammation lead to thrombosis [22]. It has been demonstrated that autophagy induction by HMGB1 is a prerequisite for neutrophil cytosolic NET generation, and blocking autophagy reverses HMGB1-induced platelet production. Further elucidation is needed regarding the mechanism of HMGB1-induced autophagosome formation on thrombus formation in ischemic stroke.

In summary, the interplay of HMGB1 release, inflammatory response, and neutrophil extracellular trap generation may be critical for stroke onset and progression, and HMGB1 may serve as a novel target for anti-inflammation and thrombosis, especially in patients with neocoronary stroke. HMGB1 is a crucial mediator of systemic inflammatory response, and observational studies from Brazil have shown that SARS-CoV-2 infection induces the upregulation of HMGB1 in patients with the most severe forms of COVID-19(Coronavirus disease 2019) [34]. The prognosis and mechanisms by which HMGB1 affect the condition of critically ill patients with new coronary strokes or underlying infectious diseases need to be further investigated.

## Receptor System of HMGB1

**Fig. 3:**
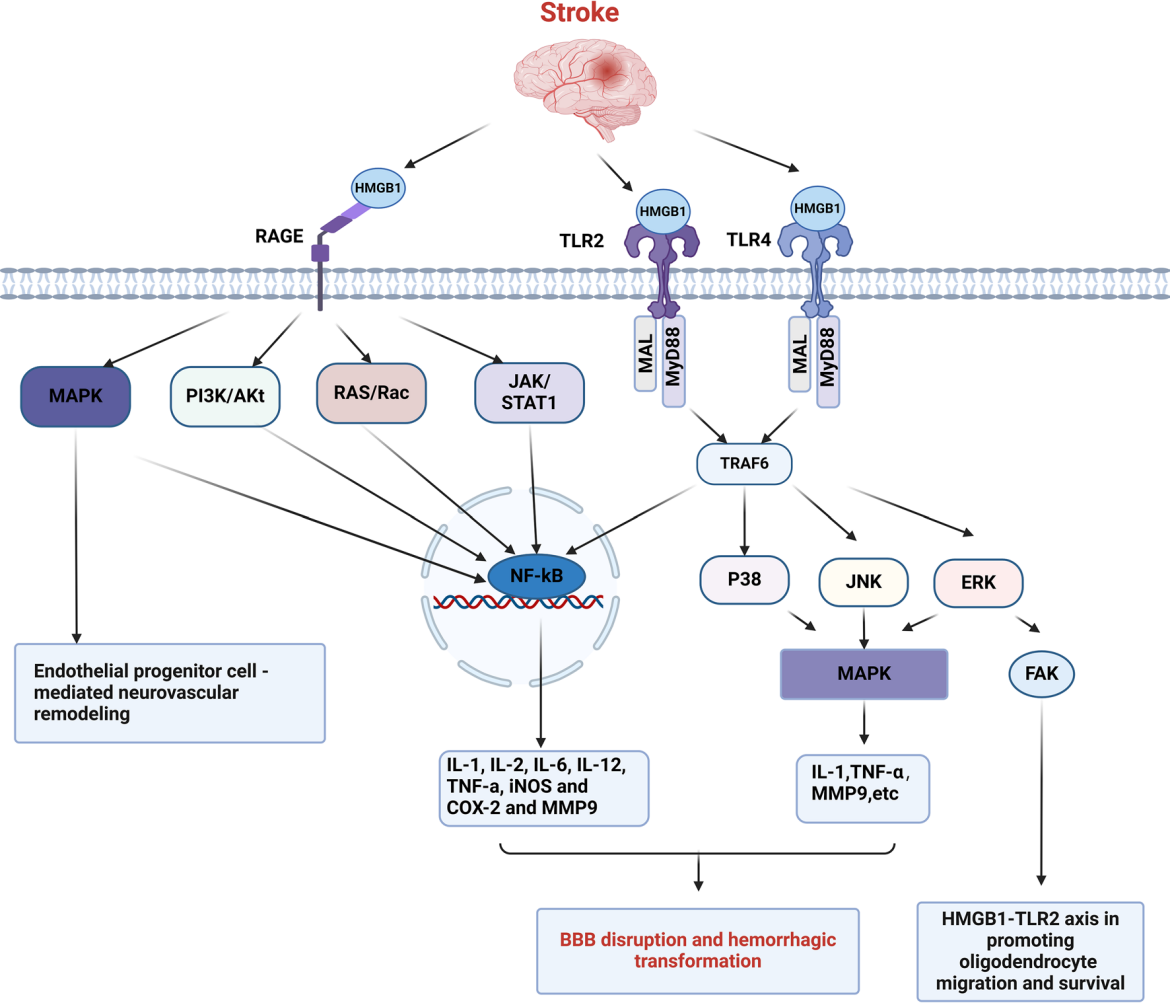
Receptor system and potential pathway of HMGB1 in stroke. HMGB1 induces BBB disruption and hemorrhagic transformation by interaction with TLR2, TLR4, and RAGE. HMGB1 also promotes MMP9 activation by binding to these receptors. The HMGB1-TLR2-ERK and HMGB1-RAGE-MAPK axis also participate in oligodendrocyte migration and neurovascular remodeling

Although there are multiple receptors for HMGB1, post-stroke HMGB1 receptor studies have centered around Toll-like receptors (TLR-2 and TLR-4) and the receptor for advanced glycosylation end products (RAGE) (Fig. 3). MMP9 is a key molecule in blood–brain barrier injury and hemorrhagic transformation, and HMGB1 is involved in MMP9 release and activation through TLR and RAGE receptors.

Toll-like receptors (TLRs) are transmembrane pattern recognition receptors (PRRs) that initiate signaling in response to multiple pathogen-associated molecular patterns (PAMPs) and danger-associated molecular patterns (DAMPs) [35]. In general, Toll-like receptor activation promotes the recruitment of adaptor proteins to activate nuclear factor NF-kB, which induces the expression of pro-inflammatory genes, inflammatory cytokines, adhesion molecules, and the activation of adaptive immunity [36]. A clinical study including patients with ischemic stroke has found that the TLR2 and TLR4 in serum were independently associated with poor prognosis after stroke and correlated with higher serum levels of IL-1β, IL6, tumor necrosis factor a (TNF-α), and the vascular cell adhesion molecule (VCAM1), and that TLR4 in serum was independently associated with lesion volume in patients with ischemic stroke [37]. HMBG1-TLR4 can regulate the activation, proliferation, migration, and survival of microglia, neural stem cells, oligodendrocytes, and neurons. At the early phase of stroke, HMBG1 mediates ischemia–reperfusion injury by activating microglia through TLR-4 signaling, such as increased NF-kB activity, NO production, transcriptional upregulation of COX-2 (cyclooxygenase2), TNF-a and IL-1β [38]. During the delayed phase, HMGB1 plays a beneficial role in stroke through TLR receptors possibly by regulating proliferation and differentiation of neural stem progenitor cells and migration of oligodendrocytes. HMGB1 affects the proliferation of neural stem cells in the subventricular zone and the differentiation of progenitor cells to neuronal progenitors through TLR4 receptors [39]. It has been found that activation of the HMGB1-TLR2 axis is associated with IκB-α degradation, ERK1/2 (extracellular signal-regulated kinase 1/2) phosphorylation, and CREB (CAMP response element binding protein) phosphorylation, promotes oligodendrocyte migration and survival, and plays a vital role in the regeneration of myelin sheaths and functional integrity after cerebral white matter strokes [40, 41]. The signaling pathways activated by TLRs are broadly classified as myeloid differentiation factor 88 (MyD88, the universal bridging protein for recruiting all TLRs except TLR3)-dependent and MyD88-non-dependent pathways [35]. HMGB1 leads to upregulation of Myd88, mediating neuronal injury, and there is a positive correlation between Myd88 expression and NFkB binding activity, pro-inflammatory cytokines TNF-a and IL-1β [42]. After HMGB1 binds to the TLRs, TLR2 or TLR4 uses MAL (MyD88 adaptor-like protein) as a bridging adaptor to recruit MyD88 and to activate the NF-κB pathway and p38 and JNK(Jun N-terminal kinase) and ERK1/2 and MAPK (mitogen-activated protein kinase) pathways [43, 44]. MyD88-independent signaling pathways are not associated with brain injury after acute cerebral ischemia/reperfusion, and several studies have suggested that Myd88-dependent pathways may be more critical than MyD88-independent pathways in stroke [38, 45].

RAGE is widely expressed in various cells, including neurons, glial cells, macrophages, neutrophils, and endothelial cells [46–48]. Under physiological conditions, RAGE expression is low in cells but increases when its ligand molecules like HMGB1 increase [44]. HMGB1-RAGE axis leads to activation of several signaling pathways like MAPK, phosphatidylinositol 3 kinase/protein kinase B (PI3K/Akt), cell division cycle 42 (Cdc42), Ras-related C3 botulinum toxin substrate (Rac), and just another kinase/signal transducer and activator of transcription 1 (JAK/STAT1)-mediated signal transduction pathways, which finally contribute to the translocation of NF-κB, triggering the expression of inflammatory cytokines and chemokines that help immune cells mature, migrate, and express surface receptors and lead to neuroinflammation [44]. After day 1 of middle cerebral artery occlusion in rats, RAGE levels were higher in the ischemic hemisphere relative to the nonischemic hemisphere [49]. HMGB1, an essential ligand for RAGE, may be involved in stroke progression through the actions of neutrophils and macrophages. After stroke, various leukocytes infiltrate the brain parenchyma, with neutrophils being the first blood-borne immune cells to invade ischemic tissue [50]. HMGB1-dependent neutrophil recruitment and activation pathway requires the functional interplay between Mac-1 and RAGE [48]. Neutrophil migration is critical for stroke prognosis and hemorrhagic transformation. Research has indicated that when tPA was treated after middle cerebral artery occlusion (MCAO), TLR4 and RAGE expression was increased in type 1 diabetic mice, and elevated TLR4 and RAGE expression was positively correlated with hemorrhagic conversion [51]. The studies suggest that the mechanisms by which HMGB1-RAGE may affect neutrophil migration and activation to mediate neuroninflammation and hemorrhagic transformation deserve further elucidation. After 4 ~ 6 h after neutrophil infiltration, monocytes adhere to the vessel wall to enter the ischemic zone and differentiate into macrophages [52]. During cerebral ischemia, macrophages consist partly of activated microglia and mostly of migratory macrophages [46]. HMGB1-RAGE signaling links necrosis to macrophage activation and may mediate post-stroke brain injury [46]. Research has indicated that HMGB1 initiates endocytosis through RAGE- and dynamin-dependent signaling, which in turn induces cellular pyroptosis [53]. It is well known that cellular pyroptosis, a mode of programmed cell death accompanied by inflammation, is closely associated with neuroinflammation and neuronal death in the early stages of stroke. Significant gaps remain in our understanding of the effect of HMGB1-RAGE axis-activated macrophages on neuroinflammation and neural focal death after ischemic stroke. Notably, plasma levels of both soluble RAGE (sRAGE) and HMGB1 were significantly increased 48 h after ischemic stroke, and sRAGE levels were an independent predictor of functional outcome 3 months after stroke [54]. Howerver, administration of recombinant sRAGE significantly improved the outcome of injury in mice, protected cultured neurons against oxygen and glucose deprivation, and ameliorated the detrimental effect of recombinant HMGB1,which indicates sRAGE may compete with cell surface RAGE for HMGB1 [15, 54].

MMPs are a group of proteases with more than 20 members that can lead to blood–brain barrier opening, brain edema, hemorrhage, and cell death, with MMP9 being the predominant form in the brain [55]. Delayed tPA (tissue plasminogen activator) treatment activates MMP9 in the ischemic brain, and activation of MMP9 subsequently disrupts extracellular matrix and tight junction proteins and mediates blood–brain barrier leakage and hemorrhagic transformation [55]. HMGB1 was significantly increased in plasma after tPA treatment in stroke patients, promoting the release of MMP9, and leading to blood–brain barrier disruption and hemorrhagic transformation [4, 56]. Studies further elucidated that HMGB1 is released from necrotic neurons and promotes MMP9 activation by binding to its receptors, including RAGE, TLR2, and TLR4 [57–59].

## Regulation of Immune Cell Phenotype and Immunoreactivity by HMGB1

**Fig. 4:**
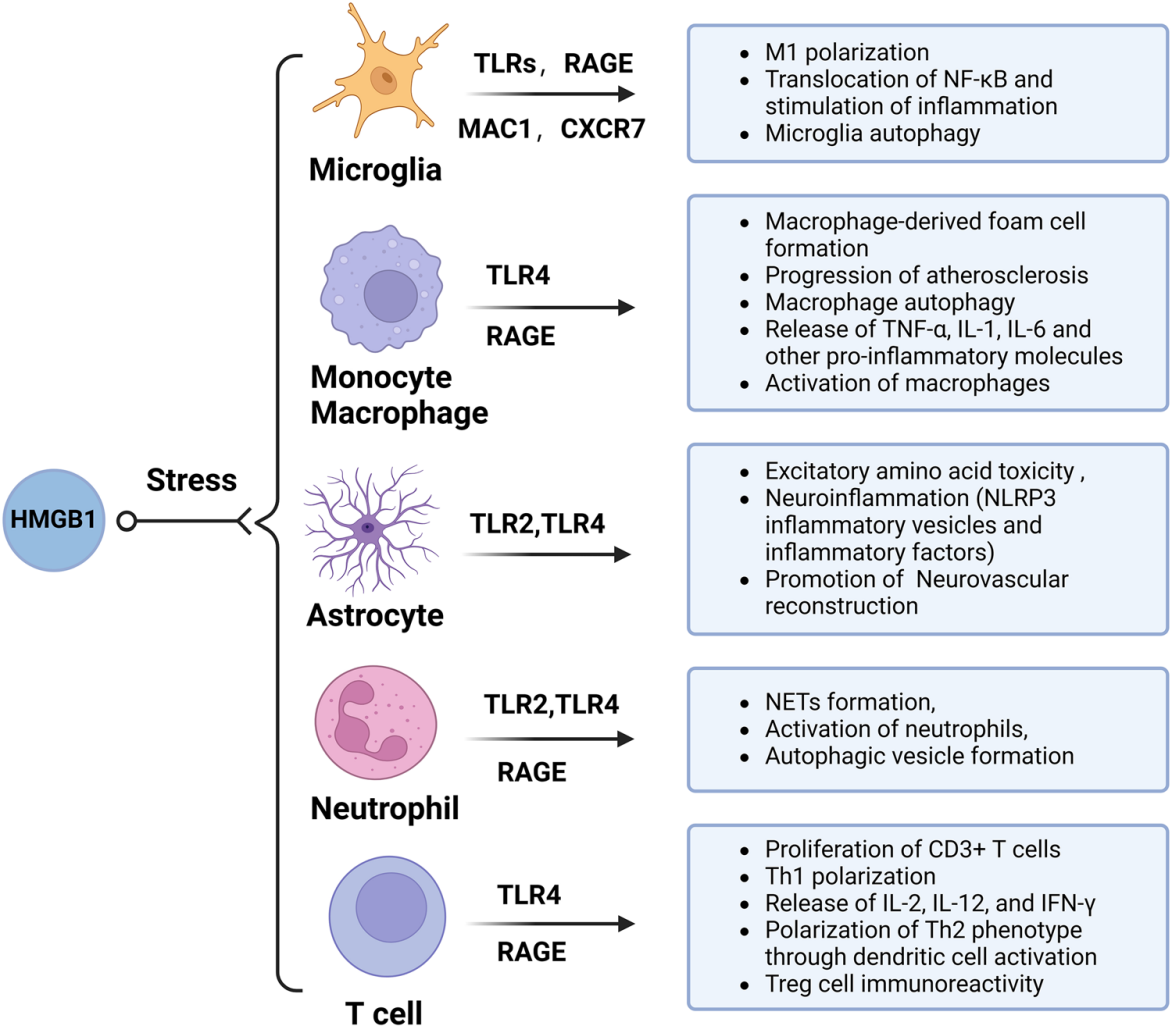
Regulation of immune cell phenotype and immunoreactivity by HMGB1. HMGB1 plays a vital role in regulating immunophenotypes and immunoreactivity in microglia, astrocytes, neutrophils, monocyte macrophages, and T cells. HMGB1 participates in neuroinflamation, excitotoxicity, autophagy, neurovascular reconstruction, NET formation, and atherosclerosis through binding with TLR, RAGE, MAC1, and CXCR receptors on these immune cell

HMGB1 plays a vital role in the regulation of immunophenotypes and immunoreactivity in microglia, astrocytes, neutrophils, monocyte macrophages, and T cells through TLR, RAGE, MAC1 (Alphambeta2), and CXCR receptors, which modulate processes such as inflammation, excitotoxicity, and autophagy (Fig. 4). The study of the immune mechanisms of HMGB1 in ischemic stroke provides new insights into neuroprotective therapy.

### Microglia

Microglia are critical cells in post-stroke immunoregulation, shifting between pro-inflammatory M1 and anti-inflammatory M2 phenotypes. M1 activation is considered a pro-inflammatory and neurotoxic state induced by Toll-like receptor (TLR) and interferon-gamma (IFN-γ) signaling pathways and is associated with an increase in pro-inflammatory mediators (IFNγ, IL-1β, TNFα, IL-6, CXCL10, etc.) in protein synthesis, ROS, and NO production, and an increase in protein hydrolases (MMP9, MMP3) that act on the extracellular matrix to cause blood–brain barrier breakdown [60, 61]. M2 activation is an immunosuppressive, pro-angiogenic, and tissue-healing state that produces anti-inflammatory cytokines (IL-10 and TGF-β), growth factors (i.e., insulin-like growth factor 1, colony-stimulating factor 1, vascular endothelial growth factor), and neurotrophic factors (glial cell-derived neurotrophic factor (GDNF) and brain-derived neurotrophic factor (BDNF)) [60]. After the acute stroke, microglia are activated within minutes, predominantly M2 cells in the early stages. Still, around the infarct zone, M2 cells are continuously recruited and converted to M1 cells, mediating neuroinflammation and disrupting the blood–brain barrier, increasing the risk of hemorrhagic transformation [4]. It has been shown that activated microglia directly engulf endothelial cells via phagocytosis and lead to vascular disintegration, ultimately disrupting the blood–brain barrier (BBB) [62].

High-mobility group protein 1 (HMGB1) is one of the essential proinflammatory mediators promoting M1 polarization of microglia, interacting and activating microglia through the receptor for advanced glycosylation end products (RAGE), Toll-like receptors (TLRs), scavenger receptor MAC1, and chemokine receptor CXCR7 [63]. HMGB1 leads to translocation of NF-κB, a significant influence factor on M1 polarization because of its role in regulating pro-inflammatory mediators and blood–brain barrier leakage, including IL-1, IL-2, IL-6, IL-12, TNF-a, iNOS (inducible nitric oxidesynthase), and COX-2 and MMP9 [44, 64]. Through selective internalization, HMGB1 is cleared by activated microglia and infiltrating monocyte macrophages, which limits cascading inflammatory injury [65]. CD36 and scavenger receptor A mediate microglia internalization [66, 67], but the unexplored pathways by which activated microglia mediate HMGB1 clearance and suppression of immune responses in stroke require attention. In addition to the internalized clearance of HMGB1, autophagy as a critical process for immune regulation in ischemic stroke needs to be further studied. Autophagic flux was induced in early OGD/R and inhibited in late OGD/R; the inhibition of autophagic flux in microglia is vital in ischemic stroke primarily by regulating microglia to M1 type and promoting inflammatory responses [68]. A study of neuroinflammation in the etiology of stress hypertension found that the HMGB1/RAGE axis mediates stress-induced impairment of mitochondrial autophagic flux and immunophenotypic switching of microglia, leading to neuroinflammatory responses such as NFκB activation and PIC release [69]. Similarly, progesterone regulates microglia activation, inflammatory response, and neuronal loss in the ischemic brain after stress by reducing HMGB1 release and NLRP3 inflammatory vesicle activation while enhancing microglia autophagy [70]. The involvement of HMGB1 in the autophagy process of microglia in stroke and as an immune target for therapeutic purposes needs to be further investigated.

Several studies have shown that glycyrrhizic acid and its hydrolyzed product glycyrrhetinic acid can reduce the size of cerebral infarction, restore motor function, inhibit M1 microglia activation, enhance M2 activation, and induce neural regeneration by inhibiting HMGB1 [63, 71]. The inhibition of HMGB1 can be an effective strategy for treating cerebral infarction injury.

### Monocyte Macrophages

Monocyte macrophages are a double-edged sword in ischemic stroke, playing an essential role in removing debris and inflammation, causing tissue damage, and promoting tissue remodeling [72]. Unlike microglia, which peak 2–3 days after ischemic stroke, monocyte-derived macrophages are not recruited in large numbers to the injured brain until 3–7 days after ischemic stroke [73, 74]. There are functional differences in monocyte subtypes in ischemic stroke, with early proinflammatory monocytes limiting ischemic stroke injury by eliminating necrotic cellular debris and maintaining cerebral microvascular stability, whereas anti-inflammatory monocytes are implicated in tissue remodeling and healing during the subacute and chronic phases of ischemic stroke [75]. Less is known about CNS border-associated macrophages, a small population of specialized macrophages localized in the choroid plexus, meninges, and perivascular spaces, which have prominent functional roles in promoting vascular leakage, leukocyte attraction and infiltration into the ischemic brain, and promoting inflammation [76].

Monocyte macrophages release HMGB1 in response to cellular injury. In turn, activated HMGB1 signaling activates macrophages to release TNF-α, IL-1, IL-6, and other pro-inflammatory molecules via TLR4 [77, 78]. H_2_O_2_ may stimulate the release of HMGB1 from macrophages and monocytes through MAPK- and crm1-dependent pathways [79]. ROS oxidized low-density lipoprotein (OxLDL), a risk factor for stroke, stimulates oxidative stress-induced secretion of HMGB1 in macrophages, and HMGB1 promotes macrophage-derived foam cell formation via the endoplasmic reticulum stress (ERS)/C/EBP-homologous protein (CHOP) pathway [80]. HMGB1 promotes monocyte-macrophage activation, amplifies inflammatory processes, and is a critical molecule in atherosclerosis. It has been shown that stroke-induced up-regulation of monocyte hexokinase 2 (Hk2) depends on HMGB1, thus mediating vascular inflammation and atherosclerosis progression after stroke [81]. However, infiltrating anti-inflammatory monocyte macrophages directly enhances the expression of the scavenger receptor Msr1 (macrophage scavenger receptor 1) via the transcription factor MAFB (MAF BZIP transcription factor B), which induces the internalized clearance of HMGB1, and impaired clearance leads to more severe inflammation and exacerbates neuronal damage [65]. In addition, HMGB1 co-localized with the autophagy protein Beclin 1 in macrophages near the necrotic core of carotid plaques, suggesting that HMGB1 may be involved in regulating macrophage autophagy [82]. Autophagy plays an essential role in the stabilization of atherosclerotic plaques by inhibiting oxidative stress, inflammation, and foam cell formation, and promoting the transformation of anti-inflammatory macrophages within the plaque [83], suggesting that HMGB1 may play a beneficial role in the progression of atherosclerosis and the prognosis of ischemic stroke through macrophage autophagy. Thus, HMGB1 as a possible target for macrophages needs to be further investigated.

### Astrocytes

Reactive astrocytes after ischemic stroke have pro-inflammatory and neuroprotective functions [84]. Reactive astrocyte proliferation occurs in the peri-infarct region, forming a glial scar to maintain CNS (central nervous system) homeostasis and isolate the lesion [85]. After undergoing reactive astrocyte proliferation, astrocytes produce and release proinflammatory mediators such as IL6, TNF-α, IL-1α, IL-1β, and IFNγ, as well as free radicals such as NO, superoxide, and peroxynitrite that can lead to neuronal death and infarct progression [86]. Astrocytes are critical for the integrity of the blood–brain barrier and may be implicated in hemorrhagic transformation. Ischemic neurons activate astrocytes to increase vascular endothelial growth factor (VEGF) production, leading to disruption of the endothelial barrier [87]. Activation of the VEGF signaling pathway is a key factor in hemorrhagic transformation after tPA, and combination treatment with tPA and anti-VEGF neutralizing antibody significantly reduced VEGF expression in blood–brain barrier, MMP9 activation, and degradation of blood–brain barrier components in a rat thromboembolic model [88].

HMGB1 plays a role in the dual action of astrocytes in ischemic stroke. HMGB1 is involved in inflammatory activation and excitatory amino acid toxicity in astrocytes. Under various stress conditions, extracellular RNA released by hypoxia/ischemia in combination with the TLR2 ligands Pam2CSK4 and HMGB1 stimulates strong activation of astrocytes and significantly promotes the expression and release of pro-inflammatory cytokines [89]. PNX-14 and the benzene derivative CD21 inhibit HMGB1-mediated activation of NLRP3 inflammatory vesicles and production of inflammatory factors such as IL-1β, IL-18, IL-6, or TNF-α, thereby exerting a neuroprotective effect [90, 91]. It has been found that cerebral ischemia/reperfusion injury activates the HMGB1/TLR4 axis by inhibiting the expression of glutamate transporter (GLAST) in primary astrocytes, thereby decreasing glutamate clearance activity and increasing excitatory amino acid-mediated ischemic injury [92]. However, some studies have demonstrated the role of HMGB1 in astrocytes in limiting brain injury and functional remodeling. Treatment with the astrocytic metabolic inhibitor fluorocitrate significantly decreased HMGB1-expressing astrocytes, suppressed neurovascular markers, and correspondingly worsened neurological outcomes [93].

### Neutrophils

Neutrophils are involved in early peripheral inflammatory infiltration and play an important role in developing ischemic stroke and hemorrhagic transformation. Increased circulating neutrophils are observed as early as 4–6 h after ischemic stroke, peaking at 1–3 days and then declining over time, with NETs detected 2–3 days after stroke [94]. Neutrophil infiltration exacerbates ischemic stroke and hemorrhagic transformation, and lower neutrophil–lymphocyte ratios are associated with independent predictors of successful reperfusion and good clinical outcome after endovascular thrombectomy, as well as a reduced risk of symptomatic cerebral hemorrhage and death [95]. The ischemic environment and the interaction of neutrophils with endothelial adhesion molecules shift the neutrophil phenotype from a protective N2 to a damaging N1 phenotype [94]. Neutrophils synthesize and secrete pro-inflammatory factors such as TNF-α, IL-1β, and IL-6 and promote the expression of MMP9, which aggravates cerebrovascular endothelial cell injury after stroke and increases the risk of hemorrhagic complications [4, 96].

NETs are composed of DNA, histones, and granule proteins, and NETs promote thrombosis and vascular occlusion by activating platelets, as well as scaffolding platelets and erythrocytes, and procoagulant molecules to promote coagulation. They are associated with reperfusion resistance in acute stroke [97–99]. It has been shown that HMGB1 is detected in all thrombus emboli and co-localizes with neutrophils and NETs, participating in NETs-mediated thrombosis and ischemic stroke development [100]. HMGB1-induced oxidative stress is associated with neutrophil activation and NET formation, and HMGB-1 influences NETs formation through its receptors TLR2, TLR4, and RAGE binding [101, 102]. In addition to this, it has been shown that activated platelets deliver HMGB1 to neutrophils and engage them in autophagy and NET generation, exacerbating thrombotic inflammatory lesions [103]. HMGB1-mediated formation and release of NETs exacerbated inflammation and neuronal damage in the ischemic brain [23]. Targeting HMGB1 is efficacious in improving clinical outcomes in stroke. Platelet depletion or platelet-specific knockdown of HMGB1 significantly reduced plasma HMGB1 and NET levels after stroke and greatly improved stroke prognosis [33]. The HMGB1/TLR4 signaling pathway inhibition promotes endogenous anti-inflammatory defenses and inhibits pro-inflammatory responses by targeting circulating neutrophils, which may be actively involved in cerebral ischemic neuroprotection [104]. In addition to this, HMGB1 increased the risk of mediating hemorrhagic transformation and reperfusion resistance after recanalization, and the effect of HMGB1 preconditioning on mechanical thrombolysis and thrombolysis needs to be further investigated.

### T Cells

T cells infiltrate significantly as early as 24 h after the middle cerebral artery occlusion model, reach peak infiltration 3–5 days after stroke, and persist for a more extended period [105, 106]. Notably, the accumulation of T cells in the post-ischemic brain is primarily driven by increased local T cell proliferation rather than T cell invasion [107]. T cells are involved in post-stroke inflammation in an antigen-dependent and late antigen-independent manner in the early phase, and the early T cell response in an antigen-independent manner is strongly correlated with the development of infarct volume [106]. After ischemic stroke, invading CD4 and CD8 T cells interact with reactive astrocytes and exhibit increased expression of T cell activation markers, proinflammatory cytokines, and corresponding transcription factors [108]. CD8 + T cells exert cytotoxicity through the release of granzyme and perforin, which form holes in target cells and induce apoptosis; on the other hand, they degrade post-stroke blood–brain barrier tight junctions through the activation of VEGF signaling, which promotes blood–brain barrier permeability [109–111]. Treg cells participate in the inflammatory response and neuroplasticity process in ischemic stroke through various mechanisms, such as secretion of anti-inflammatory factors, inhibition of pro-inflammatory factors, induction of cell lysis, involvement in microvascular dysfunction and thrombosis, production of factors promoting neuroregeneration, and modulation of microglia and macrophage polarization, and thus exert both beneficial and adverse effects in ischemic stroke [112, 113].

HMGB1 has been shown to induce the proliferation of CD3 + T cells, which can lead to Th1 polarization as well as IL-2, IL-12, and IFN-γ production, and another study showed polarization of CD4 + T cells to a Th2 phenotype through dendritic cell activation [8, 114–116].

HMGB1 plays a crucial role in T cell infiltration and proliferation, and the HMGB1 inhibitor glycyrrhizin prevents ischemia and reduces infarct size in part by inhibiting the infiltration and proliferation of T cells and their subtypes into the ischemic brain [117]. Similarly, the HMGB1 competitive inhibitory protein, HMGB1 A box, significantly inhibited the infiltration of almost all T cells, including Th17, and microglia-induced differentiation of naïve T cells to Th17 cells [118]. In addition, HMGB1 may be a critical factor in Treg cell activity through TLR4 receptor and RAGE receptor. HMGB1 promotes atherosclerosis by negatively regulating the Treg/Th17 ratio [119]. HMGB1 significantly reduced the expression of CTLA4 and Foxp3 and the secretion of IL-10 in Treg cells, and TLR4-neutralizing antibody abrogated HMGB1-induced alterations in Treg phenotype and function [120]. However, some studies have also found that HMGB1 may stimulate Treg immunosuppressive activity by binding to RAGE receptor [121, 122]. HMGB1 signaling maintains high autophagic activity, survival, and immune tolerance of Treg cells through the RAGE-ERK and mTOR pathways [123]. Taken together, the underlying mechanisms in the immune function of HMGB1 on Treg after ischemic stroke remains to be elucidated. Treg cell-derived bone-bridging proteins have been found to promote tissue-repairing microglial cell responses, thereby promoting oligodendrocyte regeneration and myelin re-formation after stroke [124]. HMGB1 has been implicated as a downstream of bone-bridging proteins in other diseases, and further discoveries are needed to determine whether HMGB1 is involved in brain repair by Treg cells.

## Role of HMGB1 in the Progression of Ischemic Stroke

HMGB1 plays complex and biphasic roles in the onset and progression of ischemic stroke. HMGB1 is released in large quantities during acute injury induced by excitotoxicity and is the main upstream inflammatory mediator within the neurovascular unit in ischemic stroke during the hyper-acute(within 24 h post-stroke) and acute phase (especially within 4–5 days post-stroke), exacerbating neuronal death and disruption of the blood–brain barrier. However, HMGB1 may exert a significant role in promoting vascular remodeling and neurological recovery during the acute (4–5 days to 1 week after stroke) and subacute(1–3 weeks after stroke), and chronic (> 3 weeks after stroke) phases. HMGB1 can be significantly translocated from the nucleus to the cytoplasm of neuronal cells at 2–4 h after ischemia–reperfusion, but the extracellular release is reduced at 12 h after reperfusion [125]. Furthermore, HMGB1 is actively secreted by activated glial and other cells 2 days after stroke, peaks on day 4, and can persist for weeks or even up to 1 month [59, 126, 127].

HMGB1 may promote ischemic stroke progression during the acute phase through excitatory amino acid toxicity, oxidative/nitrification stress, and modulation of neuroinflammation. In the rat transient MCAO model, glutamate concentration increased rapidly during cerebral ischemia, decreased during reperfusion, and increased again 1 h after reperfusion and persisted until 6 h after reperfusion; whereas anti-HMGB1 antibody prevented the sustained increase in glutamate concentration after reperfusion [125]. Post-stroke can cause a large release of reactive oxygen species in addition to an increase in excitatory amino acids. NADPH oxidase activity was significantly increased after cerebral ischemia–reperfusion, which decreased HDAC4 and HDAC5 expression and promoted apoptosis, at least to some extent, through the HMGB1 signaling pathway [128]. As a response to ischemic brain injury, cells rapidly express stress-induced heat shock proteins (Hsp), among which Hsp72 is abundantly induced in neurons in the penumbra or ischemic core and plays a protective role against oxidative stress [129, 130]. In addition to inhibiting LPS- or TNFα-induced HMGB1 release, overexpression of Hsp72 strongly inhibited HMGB1-induced expression and release of cytokines (TNFα and IL-1), which was closely related to inhibition of MAP kinases (p38, JNK, and ERK) and NF-kB pathway inhibition [131]. A recent study has found that microglial myeloperoxidase(MPO) -containing exosomes increase HOCl production in neighboring neurons and mediate disulfide HMGB1 translocation, thereby exacerbating neurological deficits in ischemic brain injury and cerebral ischemia/reperfusion injury [132]. As for HMGB1’s role in neuroinflammation, short hairpin-mediated HMGB1 knockdown or HMGB1 monoclonal antibody-mediated inhibition of HMGB1 during the hyperacute phase after stroke inhibited increased blood–brain barrier permeability, microglia activation, inflammatory factor release, and iNOS expression [126, 133].

However, key mediators of the penumbral in the acute phase mediate ischemic injury and cell death but contribute to neurovascular remodeling in the recovery phase (weeks later) [134]. In an in vivo model of cerebral ischemia, injection of HMGB1 siRNA into the ventricle at 5 d after stroke significantly suppressed endothelial progenitor cell (EPC) accumulation and peri-infarct microvessel density and exacerbated the deterioration of neurological prognosis 14 days after stroke [135]. Similarly, intraperitoneal injection of the HMGB1 inhibitor glycyrrhizin 4–14 days after tMCAO blocked the beneficial effects of improved vascular remodeling and neurobehavior after human peripheral blood-derived (hPB) -EPC transplantation [136].

### HMGB1 Promotes Atherosclerosis and Thrombosis, Leading to Stroke

**Fig. 5:**
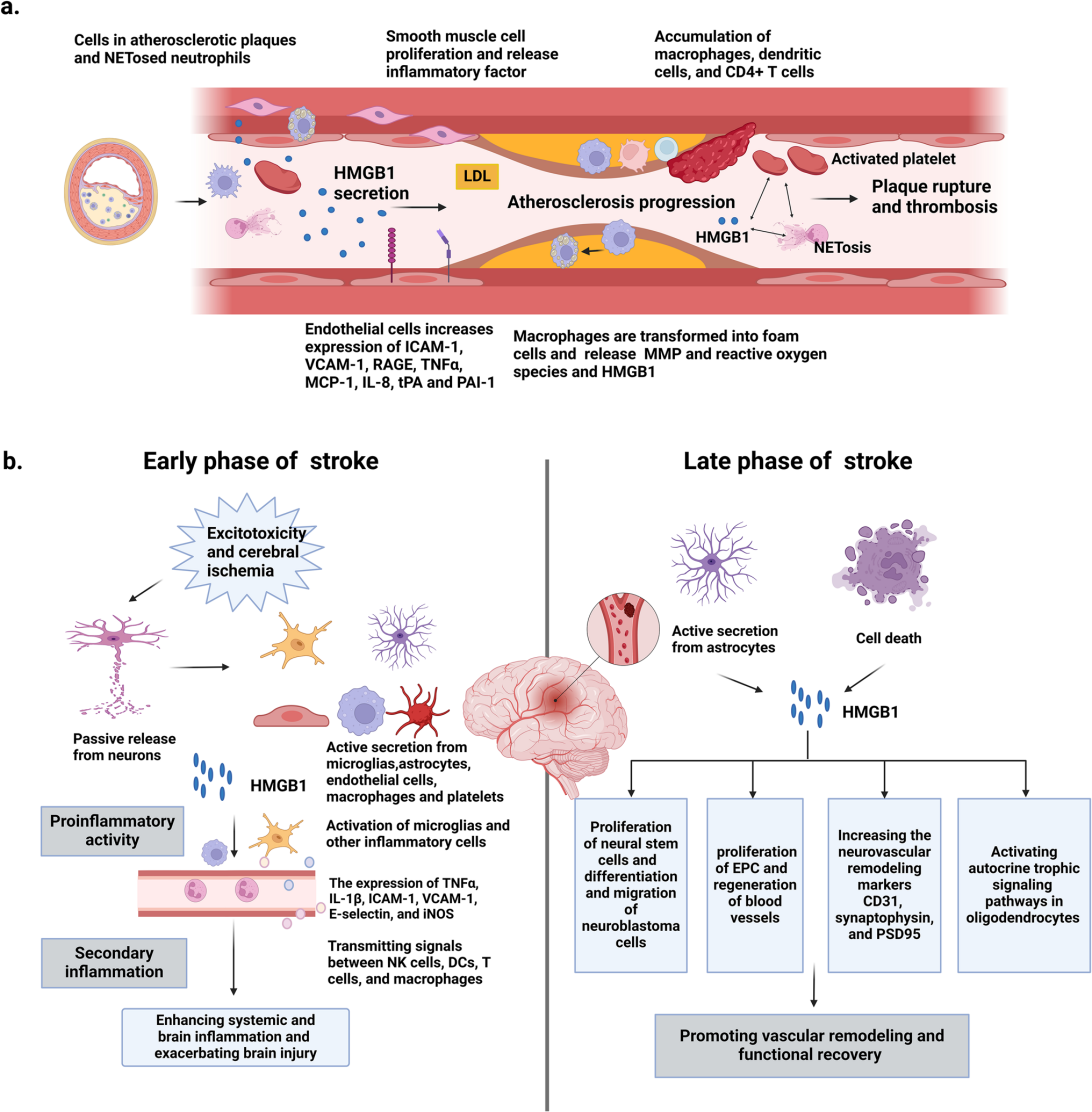
Role of HMGB1 in the progression of ischemic stroke. **a** HMGB1 promotes atherosclerosis and thrombosis, leading to stroke. HMGB1 exacerbates the progression of atherosclerosis by participating in the transport of LDL, aggregating inflammatory cells, and exacerbating the multiple inflammatory effects of HMGB1 on endothelial cells, smooth muscle cells, and macrophages. HMGB1 derived from activated platelets or NETosed neutrophils induces thrombosis and further aggravating NETosis. **b** HMGB1 promotes harmful inflammation in the early phase of ischemic stroke while plays a role in promoting vascular remodeling and functional recovery late after stroke

HMGB1 is strongly associated with atherosclerosis and thrombosis (Fig. 5), which may be involved in the development and progression of ischemic stroke. HMGB1 is involved in LDL transport through the SREBP2 (sterol regulatory element binding protein 2)-SR-BI (scavenger receptor class B type 1) axis and contributes to atherosclerosis [137]. It was found that anti-HMGB1 neutralizing antibodies led to reduced accumulation of macrophages, dendritic cells, and CD4 + T cells in atherosclerotic lesions, as well as reduced expression of the vascular cell adhesion molecule, VCAM-1, and the monocyte chemotactic protein (MCP) [137, 138]. Smooth muscle cells, endothelial cells, foam cells, macrophages, and activated platelets in atherosclerotic plaques may secrete HMGB1, exacerbating the multiple inflammatory effects of HMGB1 on endothelial cells, smooth muscle cells, and macrophages and the progression of atherosclerosis [6]. In atherosclerosis, HMGB1 promotes smooth muscle cell proliferation, migration to the intimal layer, and release of increased amounts of HMGB1 and C-reactive protein, as well as the expression of MMP2, MMP3, and MMP9 [8]. Recombinant HMGB1 activates vascular endothelial cells and increases proinflammatory expression of intercellular adhesion molecule 1 (ICAM-1), VCAM-1, RAGE, TNFα, monocyte chemotactic protein 1 (MCP-1), IL-8, tPA, and plasminogen activator inhibitor 1 [PAI-1], which is mediated by early TNFα secretion and involves the activation of stress mitogen–activated protein(MAP) kinase pathways and the transcription factors NF-kB and Sp1 [139]. HMGB1 promoted the formation and apoptosis of macrophage-derived foam cells via activation of ERS/CHOP pathway, which may be involved in atherosclerotic plaque formation and rupture [80]. After macrophages are transformed into foam cells, these cells release growth factors, cytokines, matrix metalloproteinases (MMP), reactive oxygen species, and HMGB1 and amplify inflammatory process [8]. Taken together, HMGB1 is involved in low-density lipoprotein transport, recruitment of inflammatory cells, and exacerbation of the multiple inflammatory effects of HMGB1 on endothelial cells, smooth muscle cells, and macrophages, thereby inducing the onset and progression of atherosclerosis. The progression of carotid and intracranial atherosclerosis may lead to plaque rupture and thrombosis, thus rendering the development of stroke. Oil acetic acid significantly reduces plaque HMGB1, can attenuate carotid plaque instability, and may potentially reduce the risk of ischemic stroke [140]. HMGB1 is also a critical component of the thromboembolism that leads to large-vessel occlusive stroke and is strongly associated with neutrophil and platelet counts [100]. Extracellular HMGB1 derived from activated platelets or NETosed neutrophils induces thrombosis, further aggravating NETosis and neuronal death, thereby exacerbating inflammation and subsequent damage in the ischemic brain [23, 98].

### HMGB1 as a Prognostic Marker for Cerebral Ischemia

HMGB1 has good predictive value for cerebral ischemia–reperfusion injury in patients with ischemic stroke, which correlates with leukocyte infiltration, extensive cerebral infarction, and worsened prognosis [141, 142]. Serum HMGB1 levels above 7.5 ng/mL were an independent risk factor for poor prognosis, and MMP9 activation was significantly associated with increased HMGB1 [143, 144]. HMGB1 was released in large quantities from neurons during acute injury induced by excitotoxicity and cerebral ischemia (1 h after MCAO). It was slowly but extensively induced in microglia, astrocytes, and microvascular endothelial cells of the brain after ischemia (2 days after MCAO/reperfusion), peaking on the fourth day [59]. Some studies have also suggested that the secondary release of HMGB1 is actively secreted by activated immune cells in the brain and peripheral immune systems such as microglia and invasive monocytes and macrophages in response to tissue injury, as these cells are known to actively secrete large amounts of HMGB1 upon activation [15, 27, 145]. A recent study has found that platelet depletion or platelet-specific knockdown of HMGB1 significantly reduced plasma levels after stroke and greatly improved stroke prognosis, demonstrating that platelets are also a key source of HMGB1 in the plasma during ischemic stroke brain injury [33]. Extracellular HMGB1 may act as a proinflammatory cytokine, activating microglia, and other inflammation-associated cells, stimulating the release of other cytokines and exacerbating brain injury [126]. HMGB1 participates in and amplifies the inflammatory response after ischemia, leading to activation of the inflammatory cascade through MAP kinase and NF-kB,TLR2, TLR4, and RAGE signaling, as well as the expression of TNFα, IL-1β, ICAM-1, VCAM-1, E-selectin, and iNOS in different cell types [146]. HMGB1 induces activation of glial cells and secretion of cytokines in the acute phase, then induces second infiltration of immune cells, and transmits signals between NK cells, DCs, T cells, and macrophages during the later progression, thereby amplifying the initial injury [27]. Kim et al. reported that microinjection of short hairpin RNA in the striatum within 24 h after MCAO reduced neuronal death and restricted the expression of IL-1β, COX-2, TNFα, and iNOS in rats by inhibiting the expression of HMGB1 [126]. Injection of anti-HMGB1 monoclonal antibody immediately and 6 h later after cerebral ischemia–reperfusion inhibited the increase of blood–brain barrier permeability, microglia activation, TNFα and iNOS expression, and inhibited MMP9 activity during the acute phase of stroke; conversely, intracerebroventricular injection of HMGB1 aggravated the severity of the infarction [133]. Similarly, the anti-HMGB1 antibody and HMGB1 box A (a functional antagonist of HMGB1 interaction with RAGE) significantly reduced infarct size in the MCAO mouse model [46]. Qiu et al. demonstrated that TNFa and the ICAM-1 were increased by the addition of recombinant HMGB1 in glial and endothelial cells cultured in vitro [57]. Recently, it has been found that higher HMGB1 levels in the acute phase of ischemic stroke are also associated with an increased risk of post-stroke depression and stroke-related pneumonia [147, 148]. In addition to this, HMGB1 mediates enhanced pro-inflammatory effects of lipopolysaccharides and exacerbates TLR4-dependent systemic and brain inflammation, and there is a positive feedback loop between the enhancement of LPS function by HMGB1 and the subsequent release of HMGB1 [149].

### Role of HMGB1 in Vascular and Functional Remodeling During Stroke Recovery

Interestingly, HMGB1 plays a role in promoting vascular remodeling and functional recovery late after stroke. Using a model of focal cerebral ischemia in mice, Hayakawa et al. found that reactive astrocytes expressing HMGB1 increased in the peri-infarct cortex from day 7 after cerebral ischemia in parallel with the increase of neurovascular remodeling markers CD31, synaptophysin, and PSD95 [93]. They further demonstrated that fluorocitrate had significantly decreased HMGB1-positive reactive astrocytes and neurovascular remodeling, with a corresponding deterioration in behavioral recovery in ischemic mice [93]. Vascular endothelial growth factor VEGF-A promoted the proliferation and differentiation of neural precursor cells in vitro by upregulating HMGB1 secretion from astrocytes [150]. IL-8, IL-6, and IL-1β are involved in HMGB1-mediated neuroprotection and functional recovery of astrocytes [136, 151, 152]. HMGB1-mediated activation of TLR4 is necessary to maintain the increased proliferation of neural stem cells and to promote differentiation and migration of neuroblastoma cells [39]. In addition to this, HMGB1 promotes the proliferation of EPC and the regeneration of blood vessels in ischemic areas during the chronic phase of stroke and reduces the volume of brain atrophy through the mitogen-activated protein/extracellular regulated protein kinases (MEK/ERK) pathway initiated by endothelial progenitor cell RAGE receptors [135]. Using a standard lysophosphatidylcholine injection model to induce focal demyelination of the mouse corpus callosum, Hayakawa et al. found that HMGB1 expression was upregulated in astrocytes, along with EPCs expressed pro-recovery mediators such as brain-derived neurotrophic factor and basic fibroblast growth factor within the focal white matter lesions [153]. Human peripheral blood-derived-EPC (hPB-EPC) transplantation improved neurobehavioral outcomes, reduced brain atrophy volume, and enhanced neovascularization in MCAO mice, while intraperitoneal injection of the HMGB1 inhibitor glycyrrhizin 4–14 days after tMCAO blocked the beneficial effects of hPB-EPC transplantation [136]. HMGB1/TLR2 activates autocrine trophic signaling pathways in oligodendrocytes and maintains white matter’s structural and functional integrity under ischemic conditions [41]. In an oligodendrocyte oxygen–glucose deprivation model, Choi et al. demonstrated that siRNA knockdown of HMGB1 or application of glycyrrhizin exacerbated OGD-induced oligodendrocyte death, and recombinant HMGB1 application reduced oligodendrocyte death in a TLR2-dependent manner [41]. They also confirmed inhibition of HMGB1 by glycyrrhizin amplifies demyelinating lesions in a TLR2-dependent manner with exacerbation of sensory-motor behavioral deficits in an endothelin-1-induced focal cerebral white matter stroke model [41]. Notably, some studies have found that HMGB1 may play a deleterious role in chronic cerebral ischemic white matter lesions [154, 155]. In a rat model of chronic cerebral hypoperfusion, anti-HMGB1 antibody attenuated white matter damage in the optic tract, which was associated with the downregulation of inflammatory responses characterized by glial cell activation and TLR4/NF-κB signaling downstream of HMGB1 [155].

## HMGB1 and Hemorrhagic Transformation

### HMGB1 and the Blood–Brain Barrier Injury

**Fig. 6:**
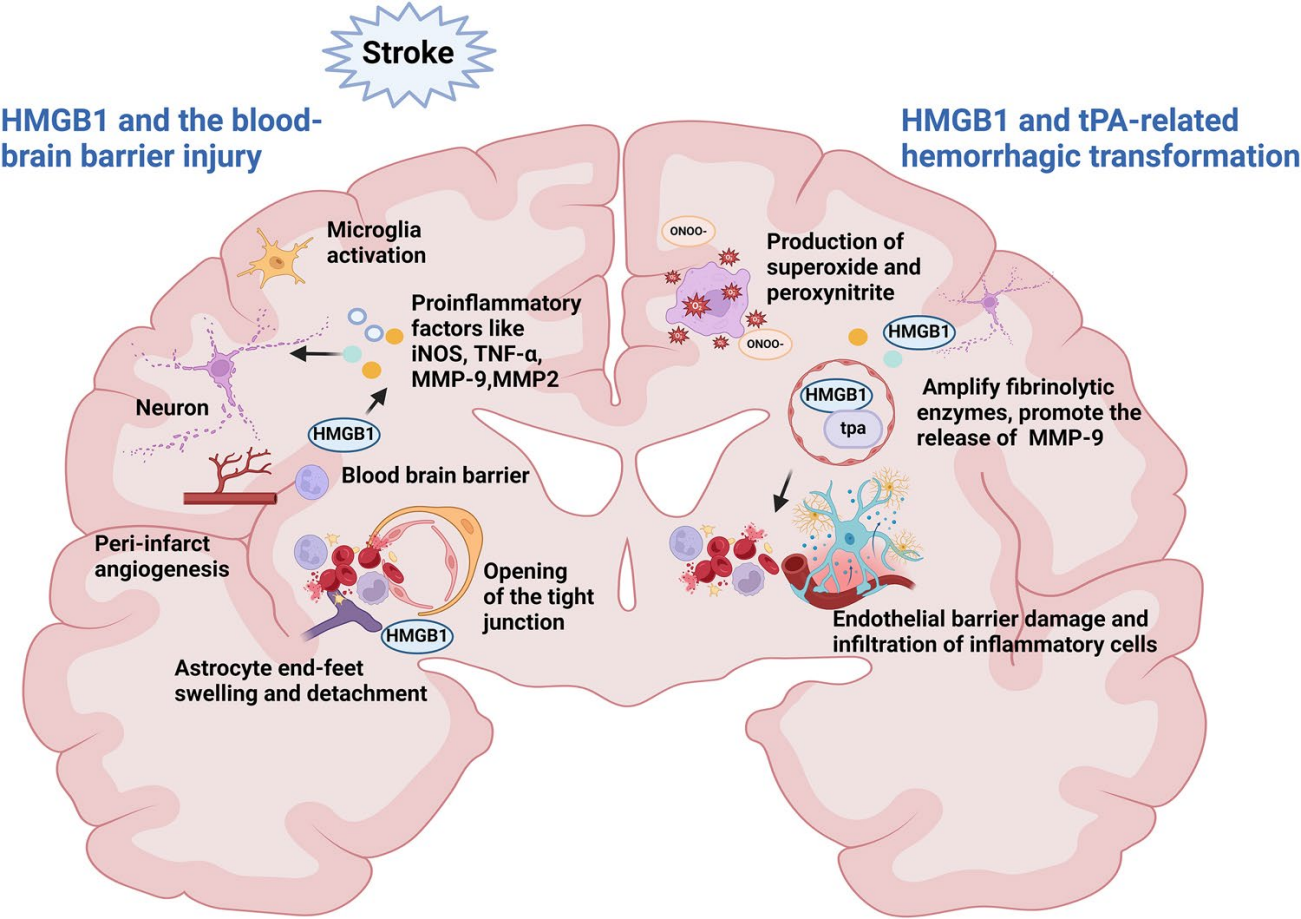
HMGB1 and hemorrhagic transformation. HMGB1 disrupts the blood–brain barrier and induces hemorrhagic transformation through direct damage to the structure of the blood–brain barrier, release of inflammatory factors, and neovascularization. In addition to this, HMGB1 binds to tissue-type plasminogen activator (tPA), which amplifies the action of plasmin, promotes the release of TNF-α and MMP9, and participates in the injury through oxidative and nitrosative stress and direct disruption of the blood–brain barrier after tPA thrombolysis

The blood–brain barrier consists of the terminal and basement membranes of vascular endothelial cells, pericytes, and astrocytes, and HMGB1 is involved in disrupting the blood–brain barrier and increasing permeability. HMGB1-mediated neuroinflammation is critical in promoting blood–brain barrier disruption and inducing hemorrhagic transformation. HMGB1 mediates the secretion of MMP9, which is involved in early blood–brain barrier degradation and plasma leakage during ischemia via pericytes [156]. Intravenous injection of anti-HMGB1 monoclonal antibody significantly protected rats from ischemia-induced blood–brain barrier disruption, which was associated with inhibiting the expression of proinflammatory factors like iNOS, TNF-α and MMP9 and microglial cell activation [133, 157]. Notoginseng leaf triterpenes also play a role in reducing blood–brain barrier and ischemia–reperfusion injury by inhibiting HMGB1 signaling, suppressing the activation of MAPKs and NF-κB, and down-regulating the concentration of inflammatory cytokines in the ischemic brain, including VCAM-1, MMP9, MMP2, and ICAM-1. Hyperglycemia activates the HMGB1-RAGE signaling pathway and induces the release of inflammatory factors and neutrophil infiltration, given that Xiao-Xu-Ming decoction inhibits RAGE-mediated neuroinflammation, blood–brain barrier disruption, and hemorrhagic transformation [158]. These studies suggest that HMGB1 can mediate blood–brain barrier damage by promoting the release of inflammatory factors such as MMP9 and the activation and infiltration of glial cells and neutrophils. HMGB1 also directly affects the cells that constitute the blood–brain barrier. Astrocyte end-feet swellings are evident around capillaries 3 h after the onset of reperfusion, and the end feet are often detached from capillary basement membranes, and tight junctions between vascular endothelial cells are dissociated (Fig. 6) [125]. These blood–brain barrier disruptions are consistent with up-regulation of pericapillary astrocyte end-foot aquaporin-4 expression, which is inhibited by anti-HMGB1 monoclonal antibodies [125]. In addition to the HMGB1 monoclonal antibody, it has now been found that histidine-rich glycoprotein (HRG) inhibits HMGB1 release and HMGB1-mediated neutrophil adhesion and endothelial barrier function to improve survival in septic mice, and further studies are needed to investigate the effect of HRG on the blood–brain barrier and as a new therapeutic option for stroke [159]. Furthermore, HMGB1 acts on target receptors on endothelial progenitor cells (EPCs) to promote peri-infarct angiogenesis, associated with ischemia/reperfusion-induced hemorrhagic transformation(HT) [135, 160].

The underlying pathophysiological mechanism of hemorrhagic transformation after mechanical thrombectomy is the disruption of the blood–brain barrier secondary to ischemic and mechanical endothelial injury, resulting in increased tissue permeability [161]. Reperfusion injury can lead to disruption of the blood–brain barrier, including endothelial cell activation, oxygen-free radical overproduction, inflammatory response, leukocyte recruitment, increased cytokine production, and edema formation [161]. In a rat cerebral venous sinus thrombosis model, mechanical thrombectomy combined with glycyrrhizin treatment inhibited the extracellular transport of HMGB1, suppressed the HMGB1-RAGE inflammatory pathway and its downstream inflammatory factors (TNF-α, IL-1β, and IL–6) and oxidative stress [162]. However, mechanisms of HMGB1 in neuroinflammation and oxidative stress leading to blood–brain barrier disruption and hemorrhagic transformation after mechanical thrombectomy in stroke models need further investigation. In addition, poor collateral circulation and recanalization failure also predispose to hemorrhagic conversion after mechanical thrombectomy. Analysis of cerebral artery thrombus emboli after mechanical thrombectomy revealed that areas containing NETs showed strong reactivity to von Willebrand factor (VWF), platelets, and HMGB1 and may make the thrombus less susceptible to dissolution [163]. Excessive NETs release exacerbates the inflammatory response, exacerbates thrombosis, disrupts the blood–brain barrier, and indicates poorer collateral circulation [164, 165]. Further research is needed to address the gaps in understanding the associated pathways between HMGB1 and NETs and their roles in hemorrhagic transformation after mechanical thrombectomy.

### HMGB1 and tPA-Related Hemorrhagic Transformation

Serum HMGB1 was increased in both patients and rats after tPA treatment, and blocking HMGB1 signaling significantly reduced neurovascular complications and tPA-induced hemorrhage transformation [160]. HMGB1 may bind to fibrinogen and tPA to amplify fibrinolytic enzymes and promote the release of MMP9, leading to the disruption of the blood–brain barrier and elevated hemorrhage transformation [4, 27]. Taken together, positive feedback activation of tPA with HMGB1 is critical for proinflammatory factor release and hemorrhagic transformation. Li et al. reported that early tPA infusion for 2 h did not induce significant hemorrhagic transformation, whereas after 4.5 h of delayed tPA treatment, significant HT was seen in the ischemic brain [160]. Meanwhile, they found that HMGB1-binding heptamer peptide (HBHP) treatment significantly down-regulated protein expression of delayed tPA-treated rat cerebral ischemia hemisphere-inducible nitric oxide synthase, COX-2, and IL-1β, which was beneficial in attenuating neurovascular complications [160]. Glycyrrhizin down-regulated the expression of NADPH oxidase and iNOS in ischemic brain tissues; inhibited the production of superoxide and peroxynitrite; decreased the expression of HMGB1, TLR2, and MMP9; and preserved type IV collagen and the tight junction protein claudin-5 thereby decreasing hemorrhagic transformation and mortality in a rat model of ischemic stroke after delayed thrombolysis (tPA infusion time as 5 h after MCAO) [56]. These studies demonstrate the important role of oxidative/nitrosative stress and immune response activation in hemorrhagic transformation after tPA thrombolysis. Reactive oxygen species and reactive nitrogen increase after reperfusion, facilitate the HMGB1 activation and release, and interact with HMGB1 downstream immune receptors such as TLR2/4 and RAGE, which is a crucial pathological mechanism to amplify cerebral ischemia–reperfusion injury [55, 166]. Significantly elevated oxygen levels during reperfusion overdrive mitochondria, increasing the ratio of oxidants to antioxidants and exacerbating oxidative stress and positive feedback injury [167]. However, the specific pathways of oxidative stress and mitochondrial dysfunction with HMGB1-mediated hemorrhagic transformation in tPA-treated hemorrhage still lack direct evidence for further in-depth studies. In addition, HMGB1 is also involved in direct endothelial barrier damage and infiltration of inflammatory cells after tPA thrombolysis. Inhibition of the hypoxia-inducible factor HIF1 protects the integrity of the blood–brain barrier by inhibiting HMGB1/TLR4/NF-κB-mediated neutrophil infiltration, thereby reducing the risk of delayed tPA (administered at 6 h after MCAO)-induced hemorrhagic transformation [168].

Hyperglycemia and hypertension are risk factors for stroke, and the role played by HMGB1 in hemorrhagic transformation after rtPA thrombolysis needs to be further elucidated. In hyperglycemic animals, tPA-induced blood–brain barrier toxicity was associated with a significant increase in TXNIP/NLRP3/IL-1β activation and an increase in HMGB-1/NF-κB/TNF-α levels [169]. HMGB-1 was also strongly associated with NLRP3 inflammatory vesicle activation, which together amplified the risk of hyperglycemia-mediated hemorrhagic transformation [169]. The expression of TLR4 and RAGE is increased in type 1 diabetic mice when treated with tPA 2 h after middle cerebral artery occlusion (MCAO) and is positively correlated with hemorrhagic transformation [51]. Thus, blocking HMGB1 signaling can help prevent complications associated with thrombolysis in ischemic stroke, especially in those with combined hyperglycemia. Hypertension is a crucial factor in hemorrhagic conversion after thrombolysis. Some studies found that hypertensive episodes affect the upregulation of RAGE in mice’s cerebral cortex and hippocampal vasculature and that telmisartan reduces blood HMGB1 levels in hemorrhagic-converted patients [170–172]. The mechanism by which the HMGB1-RAGE pathway may influence hemorrhagic conversion after thrombolysis in hypertensive patients requires further investigation.

## Therapeutic Strategies Modulating HMGB1 Signaling Pathway

### Physical Therapy

Physical therapies after ischemic stroke include remote Ischemic preconditioning, hyperbaric oxygen, and hypothermia treatment, which may be involved in inhibiting HMGB1-mediated ischemic brain injury. Inhibition of HMGB1 remote ischemic preconditioning and post-ischemic treatment reduced plasma HMGB1 levels and enhanced its neuroprotective effects against cerebral ischemia–reperfusion injury by inhibiting autophagic processes [173]. Hyperbaric oxygen ameliorated ischemic brain injury by modulating a DNA-dependent histone deacetylase SIRT1-induced HMGB1 deacetylation and inhibiting MMP9 [174]. Hypothermia inhibits infarct volume expansion and reduces IL-1β and TNF-α expression in the peri-infarct region by preventing HMGB1 release from post-ischemic neurons [175].

### Lifestyle Modification

Diet and exercise may be involved in the regulation of HMGB1 in ischemic stroke, and a low-fat diet and moderate exercise are beneficial for stroke prognosis. A chronic high-fat diet was found to exacerbate pyroptosis and necroptosis and worsen ischemic brain pathology by enhancing the HMGB1/TLR4/NF-κB signaling pathway after cerebral ischemia–reperfusion injury and leading to poor outcomes after stroke [176]. Treadmill exercise inhibits autophagy in the ischemic semi-dark band, inhibits HMGB1 translocation and binds to Beclin1, and reduces apoptosis and infarct volume [177].

### Targeted Therapy

HMGB1-targeted therapy improves the prognosis of ischemic stroke. IVIg protects neurons from HMGB1-induced neuronal cell death by regulating the expression of TLR and RAGE, the expression of the anti-apoptotic protein Bcl-2, and the phosphorylation of cell death-associated kinases, such as JNK, MAPK, and NF-κB [178]. In a 2-h MCAO rat model, administration of anti-HMGB1 monoclonal antibody immediately and 6 h later after cerebral ischemia–reperfusion had a reducing effect on infarct volume in both the cerebral cortex and striatum of rats, with reductions of 90% and 75% at 24 and 48 h, respectively [133]. In a 4-h MCAO mouse model, the anti-HMGB1 antibody reduced infarct size and swelling and improved neurological impairment and motor coordination without hemorrhagic complications compared with the IgG and delayed tPA treatment groups [179]. In addition, injection of HMGB1-binding heptamer peptide (HBHP) within 30 min of delayed tPA treatment significantly attenuated mortality, neurological scores, brain swelling, blood–brain barrier permeability, and hemorrhagic transformation in rats in a 4.5-h MCAO rat model [160]. Administration of HMGB1 A box at 1, 3, or 6 h after MCAO reduced mean infarct volume by 81.3%, 42.6%, and 30.7%, respectively, compared with untreated MCAO brains and significantly improved neurologic deficits [180]. When siRNA was administered intranasally 24, 12, or 3 h before MCAO or 1, 3, or 6 h after MCAO, HMGB1 siRNA administered 6 h after MCAO significantly reduced infarct volume by 73.8 ± 7.2% of that in the PBS-treated model [181]. These studies demonstrate that the application of HMGB1-targeted therapy during the hyperacute period after stroke may be a potential neuroprotective strategy for patients beyond the tPA treatment time window. Nanotechnology provides a new direction to improve the efficiency of therapeutic agent delivery and targeted stroke treatment. Nanomedicines can be used as carriers to enhance the delivery and transfection efficiency of anti-HMGB1 siRNAs [182]. The reactive oxygen species-sensitive nanomedicines can effectively alleviate stroke pathology by controlling the release of therapeutic agents, inhibiting the translocation of nuclear HMGB1, and modulating microglial polarization to reduce infarct volume and enhance neurogenesis [63, 182]. The cerebroprotective effects of a brain-targeted drug-loaded nanoformulation co-mediated by low-density lipoprotein receptor and neutrophil receptor are on the one hand used to improve the blood–brain barrier transport capacity and on the other hand are associated with significant down-regulation of inflammatory cytokines, neutrophil infiltration, and intracellular calcium overload, and blockade of the inflammatory signaling pathway HMGB1/TLRs/MyD88/TRIF/NF-kB [183]. Furthermore, it is noteworthy to explore the differences between blocking HMGB1 directly versus inhibiting its individual receptors. Direct blockade of HMGB1 can more completely inhibit the inflammatory response and cellular damage mediated by HMGB1 during the acute phase of stroke, but may affect other physiological processes related to HMGB1, such as DNA repair and gene transcription leading to adverse reactions or side effects. Selective inhibition of HMGB1 binding to specific receptors can more precisely modulate specific signaling pathways, thereby reducing interference with other physiological processes and providing a better safety profile; however, inhibition of a single receptor may not completely block HMGB1-mediated inflammatory responses, and therapeutic effects may be relatively weak.

### Clinical Medications and Compounds with Therapeutic Potential

Atorvastatin significantly attenuated ischemia-induced overexpression of HMGB1, RAGE, TLR4, and NF-κB, significantly improving neurological deficits and reducing cerebral edema and infarct size 24 h after stroke [184]. Melatonin and the neuropeptide PNX-14 inhibited microglia activation-mediated inflammation after stroke by suppressing the high expression and release of HMGB1 and modulating the subsequent activation of the TLR4/MyD88/NF-κB signaling pathway [185, 186]. The antitumor activity and acute myeloid leukocyte drug methylisoindigo attenuated ischemic stroke-induced brain injury by blocking the activation of NLRP3 inflammatory vesicles and modulating microglia/macrophage polarization through inhibition of the TLR4/NF-κB signaling pathway [187]. Uric acid reduced hypoxia-induced proinflammatory cytokine release and attenuated microglia activation in vivo to reduce the volume of cerebral infarction by inhibiting HMGB1-TLR4-NF-kB signaling [188]. HMGB1 has also been found to be a target for antithrombotic drugs, with salicylic acid inhibiting the chemoattractant activity of fully reduced HMGB1 as well as the increased expression of proinflammatory COX-2 induced by disulfide HMGB1. Aspirin has been found to block the release of HMGB1 from thrombin significantly- and collagen-induced activated platelets [189, 190]. Antithrombotic drugs using HMGB1 as a target in the prevention and treatment of ischemic stroke need to be further investigated. Recombinant human thrombomodulin (rhsTM) improves cerebral ischemic injury in mice without hemorrhagic complications through HMGB1 inhibition and has a longer time window to improve the prognosis of ischemic stroke potentially, and its mechanism of action needs to be further investigated [191].

### Medicinal Herbs

Medicinal herbs may be a new possibility for the treatment of ischemic stroke, and HMGB1 targeting may be an essential mechanism involved. Glycyrrhizin, a direct inhibitor of HMGB1, reduces pro-inflammatory M1-type polarization of microglia, IFN-γ mediated T-cell activity, inhibits cytochrome C release and cysteine asparaginase three activity-mediated anti-apoptotic effects, attenuates the expression levels of inflammatory and oxidative stress-related molecules such as TNF-α, iNOS, IL-1β, and IL-6 that are overexpressed in ischemic reperfusion cerebral infarction and attenuated the hemorrhagic transformation of tPA treatment by inhibiting the ONOO^−^/HMGB1/TLR2 signaling cascade [56, 71, 117, 192]. The modulation of the interaction between HMGB1, TLR4, and NF-κB by berberine should be considered a sound ischemic preconditioning strategy to reduce infarct size, neurological deficits, pathological changes, cerebral edema, and inflammatory mediators in serum and ischemic cortical tissues, and the combination of berberine and glycyrrhizin had a more substantial inhibitory effect on the HMGB1/TLR4/NF-κB as compared with either berberine alone or the glycyrrhizin pathway was more potent in inhibiting HMGB1/TLR4/NF-κB [193]. The inhibitory effect of dioscin on HMGB-1/TLR4 signaling and subsequent inhibition of inflammation showed powerful neuroprotective effects [194]. Notoginseng leaf triterpenes reduced HMGB1 expression, inhibited activation of MAPKs and NF-κB to suppress neuroinflammation, and suppressed microglia activation in the hippocampus and cortex, thereby facilitating cerebral ischemia–reperfusion-induced neuropathological changes [195]. Ginseng Rg1 inhibits HMGB1 and oxidative stress-induced activation of glial cells and MMP-induced disruption of the blood–brain barrier by regulating MAPK, thereby exerting significant neuroprotective effects against cerebral ischemic injury [196]. Salvianolic acid D inhibited nucleoplasmic translocation of HMGB1 and its downstream TLR4/MyD88/NF-κB signaling, thereby attenuating ischemia–reperfusion brain injury [197].

**Fig. 7:**
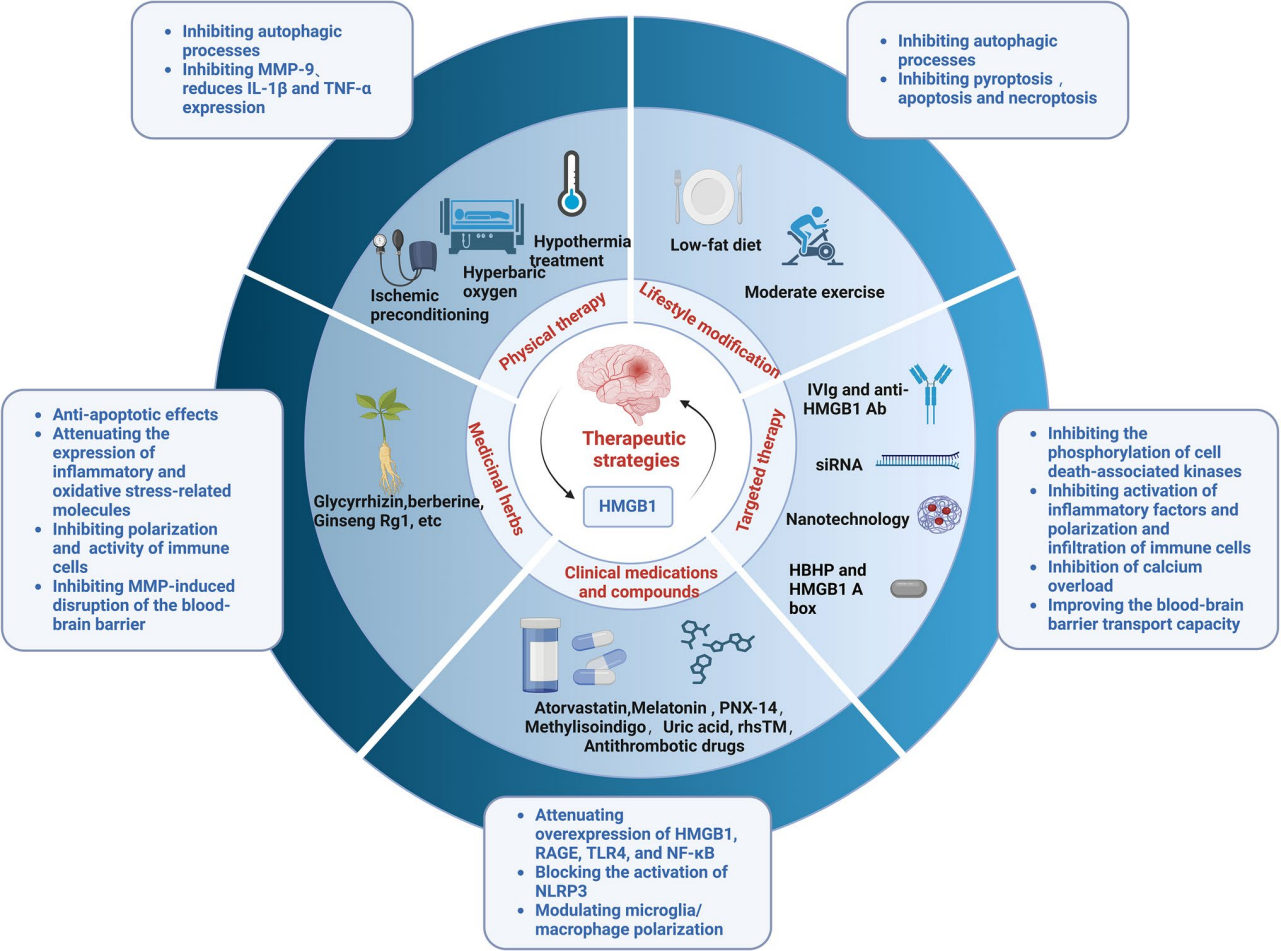
Therapeutic strategies modulating HMGB1 signaling pathway. Physical therapy, lifestyle modification, targeted therapy, clinical medications, specific compounds, medicinal herbs, or their extracts can promote neurological recovery and attenuate ischemia–reperfusion injury and infarct size after ischemic stroke through the HMGB1-mediated signaling pathway

In summary, physical therapy, lifestyle modification, targeted therapy, clinical medications, specific compounds, medicinal herbs, or their extracts can promote neurological recovery and attenuate ischemia–reperfusion injury and infarct size after ischemic stroke through the HMGB1-mediated signaling pathway (Fig. 7). However, given the complex role of HMGB1 at different times of ischemic stroke, the time window for targeting HMGB1 therapeutic modalities needs to be further investigated. HMGB1 participates in blood–brain barrier disruption and inflammatory response, and its potential as a therapeutic target for mitigating post-ischemic stroke complications such as hemorrhagic transformation after thrombolysis or infection needs to be explored.

## Conclusion

HMGB1-mediated inflammation is closely related to the development of ischemic stroke and hemorrhagic transformation, including glial cell activation, peripheral inflammatory cell infiltration, and release of inflammatory factors. In addition, HMGB1 directly affects astrocytes and vascular endothelial cells that form the blood–brain barrier and bind to tPA to amplify fibrinolytic enzymes and promote the release of MMP9, which promotes the development of hemorrhagic transformation after thrombolysis. HMGB1 promotes necroptosis and harmful inflammation in the early phase of ischemic stroke, while it plays an important role in the remodeling of the neurovascular system and the recovery of function in the late phase of stroke. Different redox states of HMGB1 regulate inflammatory responses, thrombosis, and secretion of neurotrophic factors. Immune cells and glial cells are involved in the pathogenesis of ischemia. HMGB1 plays a vital role in polarizing their different isoforms, mediating excitatory amino acid toxicity, autophagy, release of MMP9 formation of NETs, and autocrine trophic pathways. HMGB1 is an important therapeutic target and prognostic predictor of ischemic stroke and hemorrhagic transformation; however, based on the complex biphasic role of HMGB1 and the variability in treatment times between clinical and experimental models, the efficiency and safety of targeting HMGB1 in the acute treatment of cerebral infarction and long-term rehabilitation remain a major challenge in this clinical area at present.

## Data Availability

Not applicable.

## Abbreviations

BBB: Blood-brain barrier
BDNF: Brain-derived neurotrophic factor
Cdc42: Cell division cycle 42
CHOP: C/EBP-homologous protein
CNS: Central nervous system
COVID-19: Coronavirus disease 2019
COX2: Cyclooxygenase2
CREB: CAMP response element binding protein
CXCL12: C-X-C motif chemokine ligand 12
CXCR4: C-X-C chemokine receptor type 4
DAMP: Damage-associated molecular pattern
DCs: Dendritic cells
EPCs: Endothelial progenitor cells
ERK1/2: Extracellular signal-regulated kinase 1/**2**
ERS: Endoplasmic reticulum stress
GDNF: Glial cell-derived neurotrophic factor
GLAST: Glutamate transporter
HATS: Histone acetylase
HBHP: HMGB1-binding heptamer peptide
HDAC: Histone deacetylase
Hk2: Hexokinase 2
HMGB1: High-mobility group box
HRG: Histidine-rich glycoprotein
HT: Hemorrhagic transformation
hPB-EPC: Human peripheral blood-derived-EPC
ICAM-1: Intercellular adhesion molecule 1
IFN-γ: Interferon-gamma
IL: Interleukin
iNOS: Inducible nitric oxide synthase
JAK/STAT1: Just another kinase/signal transducer and activator of transcription 1
JNK: Jun N-terminal kinase
MAC1: Alphambeta2
MAFB: MAF BZIP Transcription Factor B
MAL: MyD88 adaptor-like protein
MAP: Mitogen–activated protein
MAPK: Mitogen-activated protein kinase
MCAO: Middle cerebral artery occlusion
MCP-1: Monocyte chemotactic protein 1
Msr1: Macrophage scavenger receptor 1
MMP9: Matrix metalloproteinase 9
MMPs: Matrix metalloproteinases
MyD88: Myeloid differentiation factor 88
NES: Nuclear Export Signal
NETs: Neutrophil extracellular traps
NF-kB: Nuclear factor kappa-B
NK: Natural killer cells
NLS1: Nuclear Localization Signal 1
NLS2: Nuclear Localization Signal 2
NMDA: N-Methyl-D-aspartate
OGD: Oxygen–glucose deprivation
OxLDL: Oxidized low-density lipoprotein
PAI-1: Plasminogen activator inhibitor 1
PAMPs: Pathogen-associated molecular patterns
PI3K/Akt: Phosphatidylinositol 3 kinase/protein kinase B
PRRs: Pattern recognition receptors
RAGE: Receptor for advanced glycosylation end products
RNS: Reactive nitrogen species
ROS: Reactive oxygen species
Rac: Ras-related C3 botulinum toxin substrate
RhsTM: Recombinant human thrombomodulin
rtPA: Recombinant tissue plasminogen activator
sRAGE: Soluble RAGE
SR-BI: Scavenger receptor class B type 1
SREBP2: Sterol regulatory element binding protein 2
TGF-β: Transforming growth factor beta
TLR4: Toll-like receptor 4
TLR2: Toll-like receptor 2
TLRs: Toll-like receptors
TNF-α: Tumor necrosis factor a
tPA: Tissue plasminogen activator
VCAM1: Vascular cell adhesion molecule
VEGF: Vascular endothelial growth factor
VWF: Von Willebrand factor
